# Risk factors for pulmonary complications after posterior spinal instrumentation and fusion in the treatment of congenital scoliosis: a case-control study

**DOI:** 10.1186/s12891-019-2708-8

**Published:** 2019-07-16

**Authors:** Lei Wu, Xi-nuo Zhang, Yun-sheng Wang, Yu-zeng Liu, Yong Hai

**Affiliations:** 1 Department of Orthopedics, Beijing Chaoyang Hospital, Capital Medical University, No. 8 Gongti South Rd, Beijing, 100043 China; 2 Department of Orthopedics, Beijing Haidian Hospital, Haidian section of Peking University Third Hospital, No. 29 Zhongguancun St, Beijing, 100080 China

**Keywords:** Congenital scoliosis, Pulmonary complications, Risk factors, Posterior spinal instrumentation and fusion

## Abstract

**Background:**

Although surgery prevents the progression of deformity and maintains the overall balance of the spine in congenital scoliosis (CS) patients, it is associated with a high risk of perioperative complications. Pulmonary complication is one of the most common complications. This retrospective study aimed to investigate the risk factors for pulmonary complications in CS patients after posterior spinal instrumentation and fusion.

**Methods:**

Analysis of consecutive patients who underwent posterior spinal instrumentation and fusion for congenital scoliosis was performed. Preoperative clinical data, intraoperative variables, and perioperative radiographic parameters were collected to analyze the risk factors for pulmonary complications. Patients were separated into groups with and without postoperative pulmonary complications. Potential risk factors were identified by univariate testing. Multivariate logistic regression was used to evaluate independent predictors of pulmonary complications.

**Results:**

Three hundred and twenty-three CS patients were included. Forty-five (13.9%) patients developed postoperative pulmonary complications, which included pleural effusion in 34 (75.6%) cases, pneumonia in 24 (53.3%) cases, pneumothorax in 3 (6.7%) cases, atelectasis in 4 (8.9%) cases, pulmonary edema in 2 (4.4%) cases, respiratory failure in 2 (4.4%) cases, and prolonged mechanical ventilation in 4 (8.9%) cases. The independent risk factors for development of pulmonary complications included age (Odds ratio (OR) = 1.088, *P* = 0.038), reoperation (OR = 5.150, *P* = 0.012), preoperative pulmonary disease (OR = 10.504, *P* = 0.004), correction rate (OR = 1.088, *P* = 0.001), middle thoracic screw-setting (OR = 12.690, *P* = 0.043), and thoracoplasty (OR = 5.802, *P* = 0.001). The area under the receiver operating characteristic (ROC) curve based on predicted probability of the logistic regression was 0.903.

**Conclusions:**

Age, reoperation, preoperative pulmonary disease, correction rate, middle thoracic screw-setting, and thoracoplasty were independent risk factors for pulmonary complications after posterior spinal instrumentation and fusion in CS patients.

**Electronic supplementary material:**

The online version of this article (10.1186/s12891-019-2708-8) contains supplementary material, which is available to authorized users.

## Background

Congenital scoliosis (CS) is an early-onset spinal deformity caused by vertebral abnormalities, which are classified on the basis of failures of formation, segmentation, or both [1, 2].The prevalence of CS is approximately 0.5 to 1 in every 1000 live births [3, 4]. Conservative management for CS, such as bracing and serial derotational casting, is less effective than for idiopathic scoliosis. Therefore, the surgical treatment is usually performed for CS patients with progressively worsening deformities [5]. Although scoliosis surgery prevents the progression of deformity and maintains the overall balance of the spine, it is associated with a high risk of perioperative complications [6]. Pulmonary complication is one of the most common complications because congenital spine deformities decreases not only the volume but also the function of the lungs [7–10]. However, little has been reported about postoperative pulmonary complication in CS patients. Yin et al. recently investigated the pulmonary complication in CS patients, but the sample size of their study was small and the observation variables were inadequate [11]. Therefore, we conducted this retrospective study to describe the morbidity and further try to find the risk factors of postoperative pulmonary complications after posterior spinal instrumentation and fusion surgery in CS patients.

## Methods

### Participants

The clinical data were obtained from a single-center retrospective comparative study of 323 CS patients treated by posterior spinal instrumentation and fusion at our hospital between 2011 and 2017. All the operations included in this study were performed by one surgeon (YH). This study was approved by the institutional review board following the declaration of Helsinki principles.

Inclusion criteria for this study were operatively treated CS patients with the following conditions: (1) coronal Cobb angle of thoracic, thoracolumbar, and lumbar scoliotic curves ≥40°; (2) deformities increasing in severity or predicted to have a high risk for progression (unilateral bar with a contralateral hemivertebra); (3) posterior spinal instrumentation and fusion; (4) complete preoperative and postoperative radiographic data; and (5) preoperative pulmonary function tests (PFTs) before surgery. Exclusion criteria were: (1) other kinds of scoliosis (e.g., degenerative scoliosis, ankylosing spondylitis, and spinal tuberculosis); (2) anterior approach or anterior and posterior approach; (3) growing rod; and (4) spinal trauma or tumor.

### Clinical and operative parameters

The baseline patient characteristic and demographic data were collected preoperatively (see Table 1). Clinical parameters included age, gender, body mass index (BMI), duration since diagnosis of scoliosis, preoperative pulmonary disease, operation history, preoperative white blood cell (WBC) count and proportion of neutrophil, American Society of Anesthesiologists (ASA) grade and preoperative pulmonary function (e.g., forced expiratory volume in 1 s/forced vital capacity (FEV1/FVC), residual volume/total lung capacity (RV/TLC)).

**Table 1:** Baseline clinical and perioperative characteristics of patients with or without pulmonary complication

Variables	Overall	Pulmonary complication	No pulmonary complication	*P* value
Number	323	45	278	
Age, years	17 (12–26)	26 (19–26)	15 (12–24)	< 0.001
Gender, female, no. (%)	197 (61.0%)	35 (77.8%)	162 (58.3%)	0.013
BMI, Kg/m^2^	18.4 (15.9–20.4)	17.8 (16.2–20.4)	18.4 (15.9–20.4)	0.973
Duration since diagnosis of scoliosis, years	11 (8–20)	20 (13–24)	10 (7–17)	< 0.001
Reoperation, no. (%)	41 (12.7%)	10 (22.2%)	31 (11.2%)	0.038
Preoperative pulmonary disease, no (%)	14 (4.3%)	9 (20.0%)	5 (1.8%)	< 0.001
The main bending				0.006
Thoracic scoliosis, no (%)	239 (74.0%)	42 (93.3%)	197 (70.9%)	
Thoracolumbar scoliosis, no (%)	49 (15.2%)	2 (4.4%)	47 (16.9%)	
Lumbar scoliosis, no (%)	35 (10.8%)	1 (2.2%)	34 (12.2%)	
Preoperative Cobb angle, degree	80.2 (57.3–103)	105.3 (88.3–122.3)	75 (55.2–97.7)	< 0.001
Correction rate, %	64 (57.7–71.3)	67.4 (60.1–71.3)	63.7 (57.4–71.2)	0.078
Preoperative WBC, ×10^9^/L	5.9 (5.1–7.1)	5.8 (5.0–6.9)	5.9 (5.1–7.1)	0.345
Preoperative neutrophil, %	53.3 (45.4–61.2)	55.4 (48.1–60.7)	53.1 (45.4–61.3)	0.540
ASA grade	2 (1–2)	2 (1–2)	2 (1–2)	0.886
Spinal osteotomy, no (%)	167 (51.7%)	26 (57.8%)	141 (50.7%)	0.379
No. of levels fused	12 (9–14)	13 (12–14)	12 (8–14)	< 0.001
Upper thoracic screw-setting, no (%)	208 (64.4%)	40 (88.9%)	168 (60.4%)	< 0.001
Middle thoracic screw-setting, no (%)	232 (71.8%)	44 (97.8%)	188 (67.6%)	< 0.001
Lower thoracic screw-setting, no (%)	248 (76.8%)	41 (91.1%)	207 (74.5%)	0.014
Lumbar screw-setting, no (%)	291 (90.1%)	41 (91.1%)	250 (89.9%)	0.805
Thoracoplasty, no (%)	114 (35.3%)	33 (73.3%)	81 (29.1%)	< 0.001
Operation time, min	240 (180–300)	285 (250–360)	232.5 (180–295)	< 0.001
Volume of blood transfusion, ml	400 (0–400)	500 (0–800)	0 (0–400)	< 0.001
Pulmonary function				
FEV1/FVC, %	85.4 (81.3–88.9)	83.8 (80.5–88.1)	85.5 (81.3–89.2)	0.130
RV/TLC, %	36.8 (29.9–41.8)	38.8 (33.8–43.2)	36.4 (29.6–41.6)	0.038

Value is expressed as the median (interquartile range) or number (percentage)

*Abbreviations*: *BMI* Body mass index, *WBC* White blood count, *ASA* American Society of Anesthesiologists, *No.* Number, *FEV1* Forced expiratory volume in 1 s, *FVC* Forced vital capacity, *RV* Residual volume, *TLC* Total lung capacity

Operative parameters included the instrumentation and fusion levels, spinal osteotomy levels, thoracoplasty, intra-operative blood loss, operation time, and blood transfusion.

### Radiographic measurements

Preoperative radiographs of the patients include chest X-ray, standing anterior-posterior and lateral radiographs of the whole spine, supine right and left bending radiographs, three-dimensional computed tomography (CT) reconstructions, and whole-spine magnetic resonance (MR) images. Parameters measured in the coronal plane and sagittal plane include the position and the Cobb angle of the curves, the upper and lower end vertebras of the curve, the direction of the curve, and the number of vertebras affected by curves. Postoperative radiographic data include the degree of spinal curvature correction postoperatively. All the radiographic measurements were performed independently by two spinal surgeons (LW, YSW) in order to decrease the intra-observer.

### Pulmonary complications assessment

Pulmonary complication data, which were collected postoperatively, included: pleural effusion, pneumonia, pneumothorax, atelectasis, pulmonary edema, respiratory failure, and prolonged intubation with mechanical ventilation (see Table 2). According to complication outcomes, patients were divided into two groups: with and without pulmonary complications.

**Table 2:** Numbers and percentages of pulmonary complication types among the 45 patients who developed a postoperative pulmonary complication

Pulmonary complication	Number	Frequency (%)
Pleural effusion	34	75.6
Pneumonia	24	53.3
Pneumothorax	3	6.7
Atelectasis	4	8.9
Pulmonary edema	2	4.4
Respiratory failure	2	4.4
Prolonged intubation with mechanical ventilation	4	8.9

### Statistical analysis

All the analyses were performed with the use of Stata software, version 15.1 (Stata Corp). Continuous data are expressed as means with standard deviations or as medians with interquartile ranges, depending on normality. Categorical variables were shown as proportions. In the univariate testing, categorical variables were performed using Pearson chi-square tests or Fisher exact tests where appropriate. Continuous variables were examined using Kruskal-Wallis equality-of-populations rank tests. Predictors with a *P* value of < 0.1 on univariate analysis were identified to be risk factors of pulmonary complications. The variance inflation factor (VIF) and tolerance were used to test the multicollinearity of the risk factors. A VIF > 10 or tolerance < 0.1 was identified to be significant multicollinearity. Binary logistic regression was used to determine independent risk factors of pulmonary complications among patient characteristics. The Hosmer-Lemeshow test was used to estimate the goodness of fit for the logistic regression mode. We generated a receiver operating characteristic (ROC) curve using predicted probability values from the logistic regression. A coefplot was performed to plot the regression coefficients. A nomogram was used to demonstrate the risk points and probability of independent risk factors for predicting the pulmonary complications. A *P* value of < 0.05 in 2-sided tests was statistically significant.

## Results

### Participants

Three hundred and twenty-three patients were enrolled in this study. The median operative age was 17 years, and females made up the majority of the cohort (61.0%). The median duration since diagnosis of scoliosis was 11 years. The most common main bending was thoracic scoliosis (74.0%). The median preoperative Cobb angle was 80.2 degree, and the median correction rate was 64% immediately post operation. The median number of levels fused during an operation was 12. The incidence of reoperation and preoperative pulmonary disease were 12.7 and 4.3%, respectively. The rate of thoracoplasty, spinal osteotomy, and upper thoracic, middle thoracic, lower thoracic and lumbar screw-setting was 35.3, 51.7, 64.4, 71.8, 76.8, and 90.1%, respectively. The median BMI, preoperative WBC, neutrophil, FEV1/FVC, RV/TLC, operation time, and blood transfusion volume were 18.4 Kg/m^2^, 5.9 × 10^9^/L, 53.3, 85.4, 36.8%, 240 min and 400 mL, respectively (see Table 1).

### Summary of postoperative pulmonary complications

The incidence of postoperative pulmonary complications was shown in Table 2. Forty - five (13.9%) of the 323 patients developed postoperative pulmonary complications, consisting of pleural effusion in 34 (75.6%) cases, pneumonia in 24 (53.3%) cases, pneumothorax in 3 (6.7%) cases, atelectasis in 4 (8.9%) cases, pulmonary edema in 2 (4.4%) cases, respiratory failure in 2 (4.4%) cases, and prolonged intubation with mechanical ventilation in 4 (8.9%) cases (Table 2). Twenty-four patients had one pulmonary complication, and 21 had more than one complication (14 patients with 2 pulmonary complications, 6 patients with 3 pulmonary complications, and 1 patient with 4 pulmonary complications).

### Univariate analysis

Compared with patients without postoperative pulmonary complications, patients with postoperative pulmonary complications were significantly older (*P* < 0.001), included a greater proportion of females (*P* = 0.013), and had a longer duration of symptoms (*P* < 0.001). The incidence of thoracic scoliosis (*P* = 0.006), reoperation (*P* = 0.037) and preoperative pulmonary disease (*P* < 0.001) was significantly higher in patients with postoperative pulmonary complications than in those without. The preoperative Cobb angle (P < 0.001) was lager in patients with postoperative pulmonary complications than in those without. RV/TLC value was also significantly higher in patients with postoperative pulmonary complications than in those without these complications (*P* = 0.040). More vertebral levels were fused in patients with postoperative pulmonary complications than in those without (*P* < 0.001). The rate of thoracoplasty (*P* < 0.001), and upper thoracic (*P* < 0.001), middle thoracic (*P* < 0.001), and lower thoracic screw-setting (*P* = 0.015) was significantly higher in postoperative pulmonary complications group. The operation time was longer (*P* < 0.001), and the blood transfusion volume was also higher (*P* < 0.001) in the postoperative pulmonary complications group. The Cobb angle correction rate showed a trend toward a higher risk of pulmonary complications (*P* = 0.082). No significant difference in BMI, preoperative white blood cell or neutrophil count, ASA grade, proportion of spinal osteotomy or lumbar screw-setting, FEV1/FVC was observed.

### Multivariate analysis

**Fig. 1:**
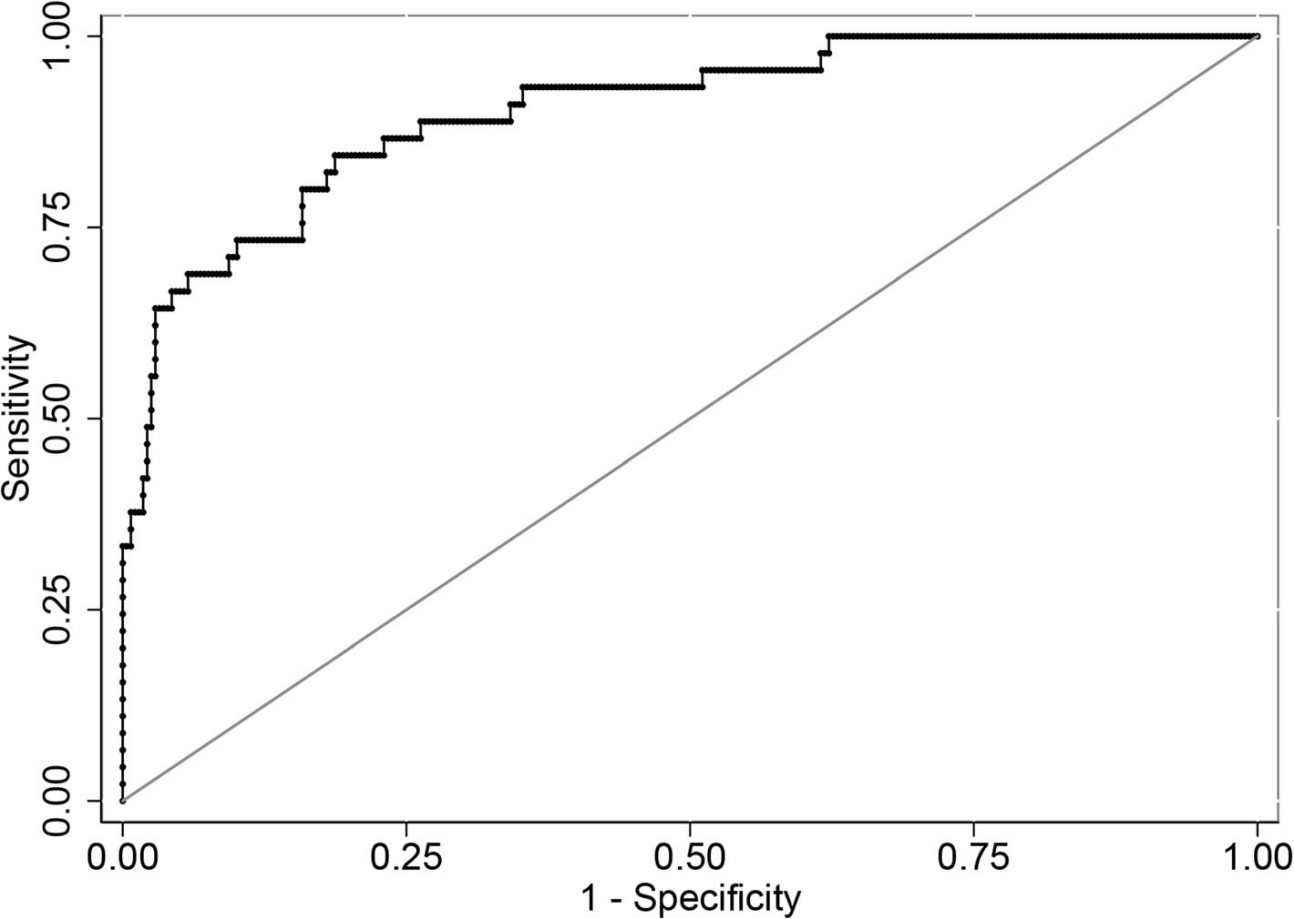
The ROC curve using predicted probability values from the logistic regression

**Fig. 2:**
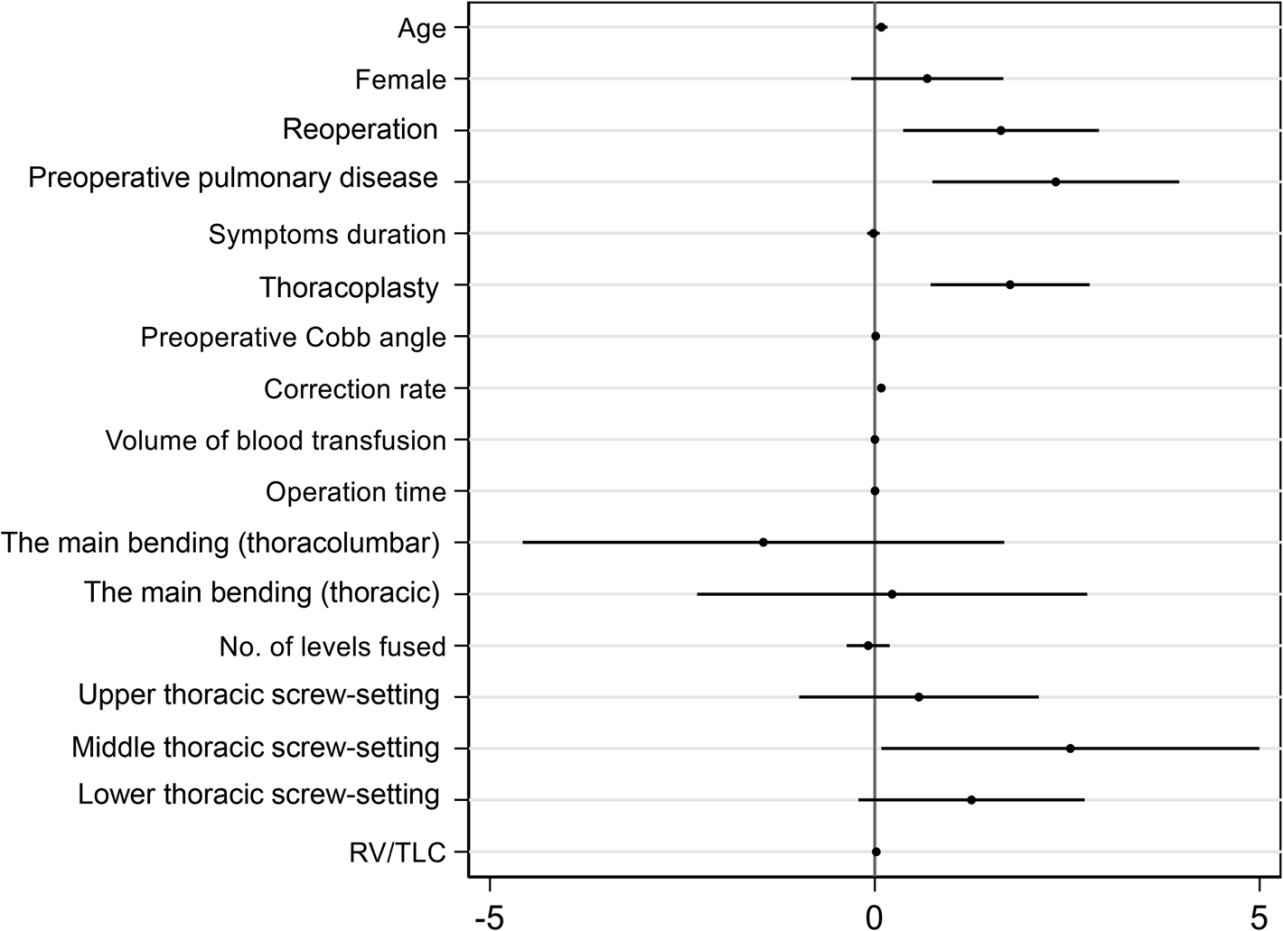
Coefplot of the Logistic regression coefficient. Abbreviation: No. number, RV residual volume, TLC total lung capacity

Variables with a *P* value of < 0.1 on univariate analysis as mentioned above were identified to be risk factors of pulmonary complications. We tested the multicollinearity of all the risk factors. The results showed that the largest VIF of all the risk factors was 3.19 and the smallest tolerance of all the risk factors was 0.31 (see Additional file 1). Therefore, we included all the risk factors in the logistic regression model, and we found that age (Odds ratio (OR) =1.085, *P* = 0.043), reoperation (OR = 5.258, *P* = 0.010), preoperative pulmonary disease (OR = 10.553, *P* = 0.004), Cobb angle correction rate (OR = 1.086, *P* = 0.002), thoracoplasty (OR = 5.601, *P* = 0.001), and middle thoracic screw-setting (OR = 12.695, *P* = 0.043) were the independent risk factors of postoperative pulmonary complications (see Table 3). The Hosmer-Lemeshow test showed the fit for the logistic regression model was good (*P* = 0.022, chi2 = 358.05). The ROC curve based on predicted probability of the logistic regression was shown in Fig. 1, and the area under the curve was 0.903 (95% CI, 0.853 to 0.952). The coefplot of the regression coefficients was shown in Fig. 2.

**Table 3:** Logistic regression of pulmonary complication and clinical variables

Variables	OR	*P* value	95% CI
Age, years	1.088	0.038	1.005–1.179
Reoperation, no. (%)	5.150	0.012	1.443–18.384
Preoperative pulmonary disease, no (%)	10.504	0.004	2.114–52.198
Correction rate, %	1.088	0.001	1.034–1.145
Middle thoracic screw-setting, no (%)	12.690	0.043	1.089–147.945
Thoracoplasty, no (%)	5.802	0.001	2.065–16.303

*Abbreviations*: *No.* Number, *RV* Residual volume, *TLC* Total lung capacity

### Nomogram

**Fig. 3:**
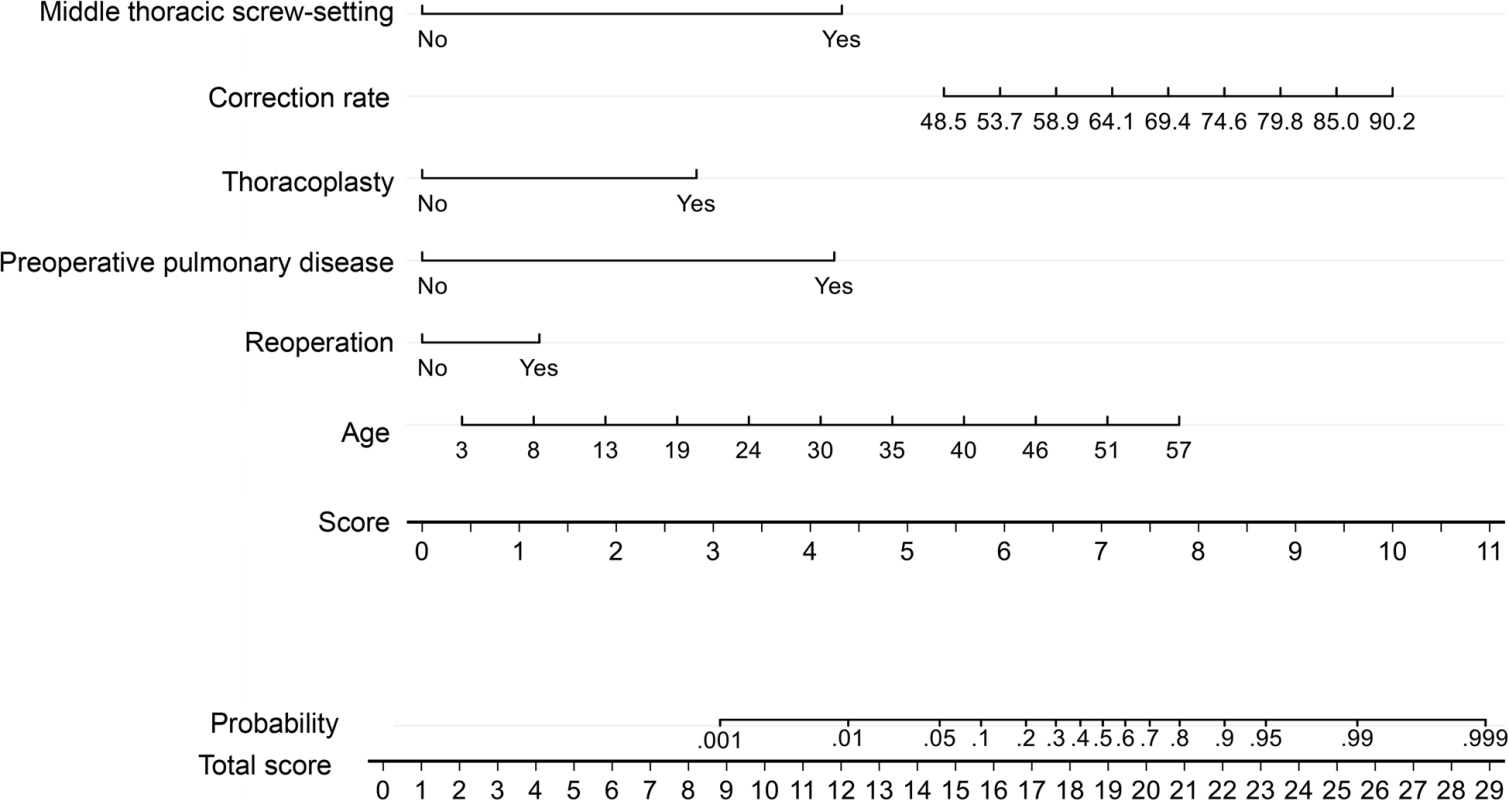
Nomogram for pulmonary complication using the independent prognostic factors

Through the logistic regression model, we built a prognostic nomogram incorporating the above independent prognostic factors for visualization and facilitating clinical practice as shown in Fig. 3.

## Discussion

Congenital scoliosis (CS) may result in thoracic deformity that limits normal respiration and lung growth [3]. Many patients with CS have progressive restrictive lung disease, which increases the risks of pulmonary complications after surgical correction [12]. Pulmonary complications are often cited as the most frequent problems after correction of scoliosis; the identification of risk factors for pulmonary complications in CS patients improves surgical safety. In our study, the incidence of postoperative pulmonary complications after posterior spinal instrumentation and fusion in the treatment of CS is 13.9%. Age, preoperative pulmonary disease, reoperation, correction rate, middle thoracic screw-setting, and thoracoplasty are independent predictive factors for postoperative pulmonary complications in CS patients.

Our study showed that age was an independent risk factor for the development of postoperative pulmonary complications. Our result is consistent with that of previous studies [12–14]. Yuan et al. [12] reported that older patients (*>* 13 years) required prolonged postoperative mechanical ventilation after scoliosis repair surgery. Patil et al. [13] found that in idiopathic scoliosis patients, older ones (*>* 18 years) were more likely to develop postoperative complications that included pulmonary problems. Shaw et al. [14] concluded that increasing age was correlated with higher rates of major short-term complications (including pulmonary complications) in adult scoliosis surgery. A possible reason for the association of age with pulmonary complications is that restrictive ventilatory impairment and pulmonary function damage caused by scoliosis are progressive with age.

In our study, CS patients with pulmonary comorbidity were more likely to have postoperative pulmonary complications than those without pulmonary comorbidity. Pulmonary comorbidity in CS patients, which was probably associated with impaired lung function, increased the risk of pulmonary complications following surgical correction. Patil et al. [13] reported that patients with preoperative pulmonary comorbidities were more likely to develop pulmonary complications after surgical correction of idiopathic scoliosis. Toll et al. [15] also identified pulmonary comorbidity in neuromuscular scoliosis patients as a risk factor for perioperative infection following surgical deformity correction. On the other side, Zhang et al. [16] found that preoperative pulmonary symptoms usually predicted abnormal results of pulmonary function tests but had no correlation with postoperative pulmonary complications in the surgical treatment of scoliosis. Although the results of previous studies were controversial, knowledge of pulmonary comorbidity provided the identification of a patient with high risk for postoperative pulmonary complications.

Our results demonstrated that the history of previous operations was an independent risk factor for the development of postoperative pulmonary complications in CS patients and more attention should be paid to reoperation patients. Sansur et al. [17] reported that patients with a previous history of spinal surgery were significantly more likely to have complications than patients undergoing initial corrections through adult scoliosis surgery. Toll et al. [15] showed that having previous operations had a strong association with infectious complications, including pulmonary ones.

According to our logistic regression analysis, the correction rate is an independent risk factor for the development of postoperative pulmonary complications. However, little has been reported about correction rate as an independent risk factor for pulmonary complications in CS patients. In our univariate analysis study, the preoperative Cobb angle (*P* < 0.001) is also larger in patients with postoperative pulmonary complications than in those without. After the test of the collinearity diagnostics, we included all the risk factors in the logistic regression model. We found that the Cobb angle correction rate (OR = 1.086, *P* = 0.002) was an independent risk factor of postoperative pulmonary complications, but, the preoperative Cobb angle (OR = 1.012, *P* = 0.246) was not. In other words, the Cobb angle correction rate is a better predictor of postoperative pulmonary complications than the preoperative Cobb angle. A possible explanation is that the degree of Cobb angle correction is more difficult for the lung and pleura to accommodate than the preoperative Cobb angle.

It is well known that thoracic pedicle screw fixation which has excellent deformity correction and a high margin of safety, is a reliable method of treating spinal deformities [18]. Yet, pulmonary complications have also been reported after thoracic pedicle screw fixation [18–20]. In our univariate analyses, the rates of upper, middle, and lower thoracic screw-settings were all significantly higher in the postoperative pulmonary complications group than in the group without complications, while the rate of lumbar screw-setting showed no significant difference between the two groups. However, in multivariate analysis, only middle thoracic screw-setting was the independent predictive factor for postoperative pulmonary complications. This may be attributed to the anatomic characteristics of the middle thoracic spine, which is located at the apex of the kyphosis of the thoracic spine. Therefore, because the middle thoracic screw-setting approach is more likely to cause injury to the lungs and pleura, greater care must be given to this procedure.

Thoracoplasty by means of multiple rib resections is used to treat the rib cage deformity in thoracic scoliosis and has been regarded as an important factor for the patient’s satisfaction [21]. Thoracoplasty in combination with spine fusion is an established method to address the rib cage deformity in scoliosis surgery [22]. With respect to the impact of postoperative pulmonary complications on thoracoplasty after scoliotic surgery, Liang et al. [23] demonstrated that performance of a thoracoplasty was the only risk factor for postoperative pulmonary complications in patients undergoing posterior spinal fusion. However, Suk et al. [21] reported that thoracoplasty showed satisfactory clinical outcomes without pulmonary function compromise in the treatment of thoracic adolescent idiopathic scoliosis. Hod-Feins et al. [8] suggested that thoracoplasty could be added whenever indicated because thoracoplasty did not correlate with postoperative pulmonary complications. In our study, CS patients who underwent thoracoplasty were more likely to have postoperative pulmonary complications than those who did not. According to our logistic regression analysis, thoracoplasty is an independent predictive factor-but no the only one for postoperative pulmonary complications in CS patients. We could use nomogram to evaluate the risk of postoperative pulmonary complications before surgery; rather than adding thoracoplasty whenever indicated, we can instead perform it in one-stage with posterior spinal instrumentation and fusion surgery.

This study has several limitations. First, this is a single center retrospective study. Second, we did not consider the postoperative pulmonary function and the outcomes of pulmonary complications, such as mechanical ventilation time, postoperative analgesia, hospital cost, and mortality. Third, we derived six independent risk factors of postoperative pulmonary complications, and built a nomogram for visualization and facilitating clinical practice. However, we did not validate the nomogram in a new database. A prospective, multicenter study is needed to address these issues and validate our findings.

## Conclusions

We conclude that it is important for surgeons to predict postoperative pulmonary complications in CS patients before surgery. Logistic regression analysis shows that the independent risk factors to develop pulmonary complications after posterior spinal instrumentation and fusion in CS patients are age, preoperative pulmonary disease, reoperation, correction rate, middle thoracic screw-setting, and thoracoplasty.

## Additional file

Additional file 1: Multicollinearity test of risk factors of pulmonary complications (DOCX 15 kb)

## Data Availability

The datasets used and analyzed during the current study are available from the corresponding author on reasonable request.

## Abbreviations

BMI: Body mass index
CS: Congenital scoliosis
CT: Computed tomography
FEV1/FVC: Forced expiratory volume in 1 s/forced vital capacity
MR: Magnetic resonance
OR: Odds ratio
PFTs: Pulmonary function tests
ROC: Receiver operating characteristic
RV/TLC: Residual volume/total lung capacity
VIF: Variance inflation factor
WBC: White blood cell
