# Supplementary material for: Risk factors for pulmonary complications after posterior spinal instrumentation and fusion in the treatment of congenital scoliosis: a case-control study
Source: BMC Musculoskelet Disord. 2019 Jul 16;20:331. doi: 10.1186/s12891-019-2708-8 (PMC6631870; doi:10.1186/s12891-019-2708-8)
Supplement: Supplementary file 1 — Multicollinearity test of risk factors of pulmonary complications (DOCX 15 kb) [file 12891_2019_2708_MOESM1_ESM.docx]

**Additional file 1** Multicollinearity test of risk factors of pulmonary complications

| Risk factors | VIF | Tolerance |
| --- | --- | --- |
| Age | 2.84 | 0.35 |
| Gender | 1.09 | 0.92 |
| Symptoms duration | 2.81 | 0.36 |
| Reoperation | 1.10 | 0.91 |
| Preoperative pulmonary disease | 1.09 | 0.92 |
| The main bending | 1.82 | 0.55 |
| Preoperative Cobb angle | 1.99 | 0.50 |
| Correction rate | 1.09 | 0.92 |
| No. of levels fused | 3.19 | 0.31 |
| Upper thoracic screw-setting | 2.60 | 0.38 |
| Middle thoracic screw-setting | 1.68 | 0.59 |
| Lower thoracic screw-setting | 1.32 | 0.76 |
| Thoracoplasty | 1.46 | 0.68 |
| Operation time | 2.24 | 0.45 |
| Volume of blood transfusion | 1.43 | 0.70 |
| RV/TLC | 1.11 | 0.90 |

Abbreviation: VIF variance inflation factor, No. number, RV residual volume, TLC total lung capacity.
